# A Chromosome Level Genome Assembly of a Winter Turnip Rape (*Brassica rapa L*.) to Explore the Genetic Basis of Cold Tolerance

**DOI:** 10.3389/fpls.2022.936958

**Published:** 2022-07-15

**Authors:** Junyan Wu, Xin-Dong Xu, Lijun Liu, Li Ma, Yuanyuan Pu, Wangtian Wang, Xue-Yang Hua, Jia-Ming Song, Kede Liu, Guangyuan Lu, Yan Fang, Xuecai Li, Wancang Sun

**Affiliations:** ^1^College of Agronomy, Gansu Agricultural University, Lanzhou, China; ^2^State Key Laboratory of Aridland Crop Science, Gansu Agricultural University, Lanzhou, China; ^3^State Key Laboratory for Conservation and Utilization of Subtropical Agro-bioresources, College of Life Science and Technology, Guangxi University, Nanning, China; ^4^National Key Laboratory of Crop Genetic Improvement, Huazhong Agricultural University, Wuhan, China; ^5^Oil Crops Research Institute, Chinese Academy of Agricultural Sciences, Wuhan, China

**Keywords:** *Brassica rapa*, graph-based pan-genome, structural variation (SV), cold tolerance, physiological characteristics

## Abstract

Winter rapeseed (*Brassica rapa L*.) is an important overwintering oilseed crop that is widely planted in northwest China and suffers chronic low temperatures in winter. So the cold stress becomes one of the major constraints that limit its production. The currently existing genomes limit the understanding of the cold-tolerant genetic basis of rapeseed. Here we assembled a high-quality long-read genome of *B. rapa* “Longyou-7” cultivar, which has a cold-tolerant phenotype, and constructed a graph-based pan-genome to detect the structural variations within homologs of currently reported cold-tolerant related genes in the “Longyou-7” genome, which provides an additional elucidation of the cold-tolerant genetic basis of “Longyou-7” cultivar and promotes the development of cold-tolerant breeding in *B. rapa*.

## Background

*Brassica rapa* is a crop species of nutritional and economic importance. It is cultivated worldwide as oil and vegetable crops. It belongs to the genus *Brassica*, tribe *Brassiceae* of the family *Brassicaceae*. *B. rapa* (AA, 2n = 20) is one of the three diploid ancestors of *B. napus* (AACC, 2n = 38) and *B. juncea* (AABB, 2n = 36). During diversification, *B. rapa* formed different subspecies and morphotypes, including turnips, leafy greens, such as bok choy and Chinese cabbage, and oilseed crops, such as turnip rape and yellow sarsons (Gómez-Campo and Prakash, 1999; Prakash et al., 2011). Many of the *B. rapa* crops are annual, but turnips, some turnip rape cultivars, and some Chinese cabbage are biennial and require vernalization to flower (Zhao et al., 2007). The oleiferous form, namely *B. rapa* ssp. *oleifera*, or turnip rape, is the third most important *Brassica* oilseed crop after *B. napus* and *B. juncea*, and is widely grown in China, Canada, India, and northern Europe (Ramchiary and Lim, 2011). Before the introduction of *B. napus* into China in the 1930's, winter turnip rape was the major Brassica oilseed crop in the provinces of South China and along the Yangtse River (Wang, 2010), but it hardly survives in North China, including the Loess Plateau and the large areas North to the Yellow River due to the prolonged low temperature and dry weather in winter. In recent years, several winter turnip varieties including “Longyou-6” (LY6) and “Longyou-7” (LY7) with strong freeze tolerance were released and extensively grown in these regions, which greatly enlarged the area of oilseed rape production in China. The varieties are sowed in late August or early September. Old leaves were beginning to turn yellow from October and all leaves withered in late November as the temperature below −10°C and covered the surface of the land, but the shoot apexes keep alive across the winter. New leaves sprout out from the alive shoot apexes next March when the temperature turns warm. The plantation of these super freeze-resistant varieties not only changes the farming system from single cropping to one and a half or double cropping per year but also increases the land surface coverage during winter and prevents soil erosion of naked land in North China by the strong wind.

A high-quality reference genome is a valuable resource for genetic and genomic studies. The *B. rapa* genome was the first to be sequenced among the *Brassica* species (Wang et al., 2011). The multi-national *B. rapa* Genome Sequencing Project (BrGSP) was launched in 2003, which aimed to obtain the genome sequence of Chinese cabbage accession “Chiifu-401-42” using a BAC-by-BAC strategy (Trick et al., 2007). The first released *B. rapa* cv. “Chiifu-401-42” draft reference genome, v1.5, was assembled using a whole-genome shotgun strategy with Illumina short reads (Wang et al., 2011). However, the first *B. rapa* genome assembly (version 1.5) is only about 283.8 Mb, 58.52% of the estimated genome size (485 Mb) (Wang et al., 2011). The *B. rapa* genome v2.0 was *de novo* assembled with an additional 76G Illumina paired-end reads (≈156×) and 6.5G PacBio single-molecule data (≈13×) (Cai et al., 2017). It was updated to the *B. rapa* genome v2.5 after improving the scaffold order (http://brassicadb.org/brad/datasets/pub/Genomes/Brassica_rapa/V2.0/V2.5/). However, due to the relatively recent whole genome triplication, highly repeated sequences, and complicated centromeric regions, the early three versions of reference genomes assembled mainly using short reads are highly fragmented and contain thousands of discrete contigs and a large number of misassemblies. The inaccuracy in assembly and the low contiguity of these draft assemblies have largely hindered their applications in both genomic and genetic studies of *B. rapa* and other related Brassica species. More recently, a significantly improved *B. rapa* draft genome (v3.0) was assembled using single-molecule PacBio sequencing, optical mapping, and chromosome conformation capture technologies (Hi-C) (Zhang et al., 2018). Relative to the previous reference genomes, the v3.0 assembly reached a contig N50 size of 1.45 Mb, representing a ~30-fold improvement of contiguity.

*B. rapa* is a highly diverse and widely cultivated crop species worldwide. The “Chiifu-401-42” reference genome only is not sufficient to capture all or even most of the variants and can hardly satisfy the needs of subsequent functional genomics research and molecular breeding of *B. rapa*. Multiple high-quality reference genomes representing different morphotypes and ecotypes are necessary for a better understanding of the genome structure and genetic basis of morphotype and ecotype differentiation in *B. rapa*. Using the long-read sequencing technologies such as Oxford Nanopore Technology (ONT) and Pacific Biosciences (PacBio), 22 *B. rapa* varieties including morphotypes of Chinese cabbage, turnip, oilseed, taicai, mizuna, and pak choi (pak choi, wutacai, caixin) have been sequenced so far and assembled in high-quality in terms of continuity and completeness of repetitive regions (Belser et al., 2018; Cai et al., 2021; Li et al., 2021). Based on 18 *B. rapa* genomes, structural variations (SVs) were identified and an integrated graph-based pan-genome was constructed. Based on the pan-genome, SVs were genotyped in 524 *B. rapa* genomes, and SVs involved in leafy head domestication were identified (Cai et al., 2021).

However, among the 22 accessions sequenced, none has been indicated to be winter turnip rape. These genomes may not be suitable for elucidating candidate genes associated with cold resistance. To obtain a high-quality reference genome for the identification of genes involved in cold resistance, in this study, we assembled a high-quality genome of “LY7” using PacBio HIFI reads and confirmed the whole genome duplication (WGD) event that occurred in the genome of *B. rapa* (Wang et al., 2011). Combining the genomes of “LY7” and the other 22 *B. rapa* accessions, we constructed a graph-based pan-genome. Joint analysis of pan-genome and RNA-seq data identified two genes, *HDG1* and *BrANS3*, which may be associated with cold tolerance. The reference genome of “LY7” and the graph-based genome will be useful for the identification of cold resistance genes through map-based gene cloning and genome-wide association studies in the future.

## Results

### Morphological Characteristics of *B. rapa* “Longyou-7”

**Figure 1:**
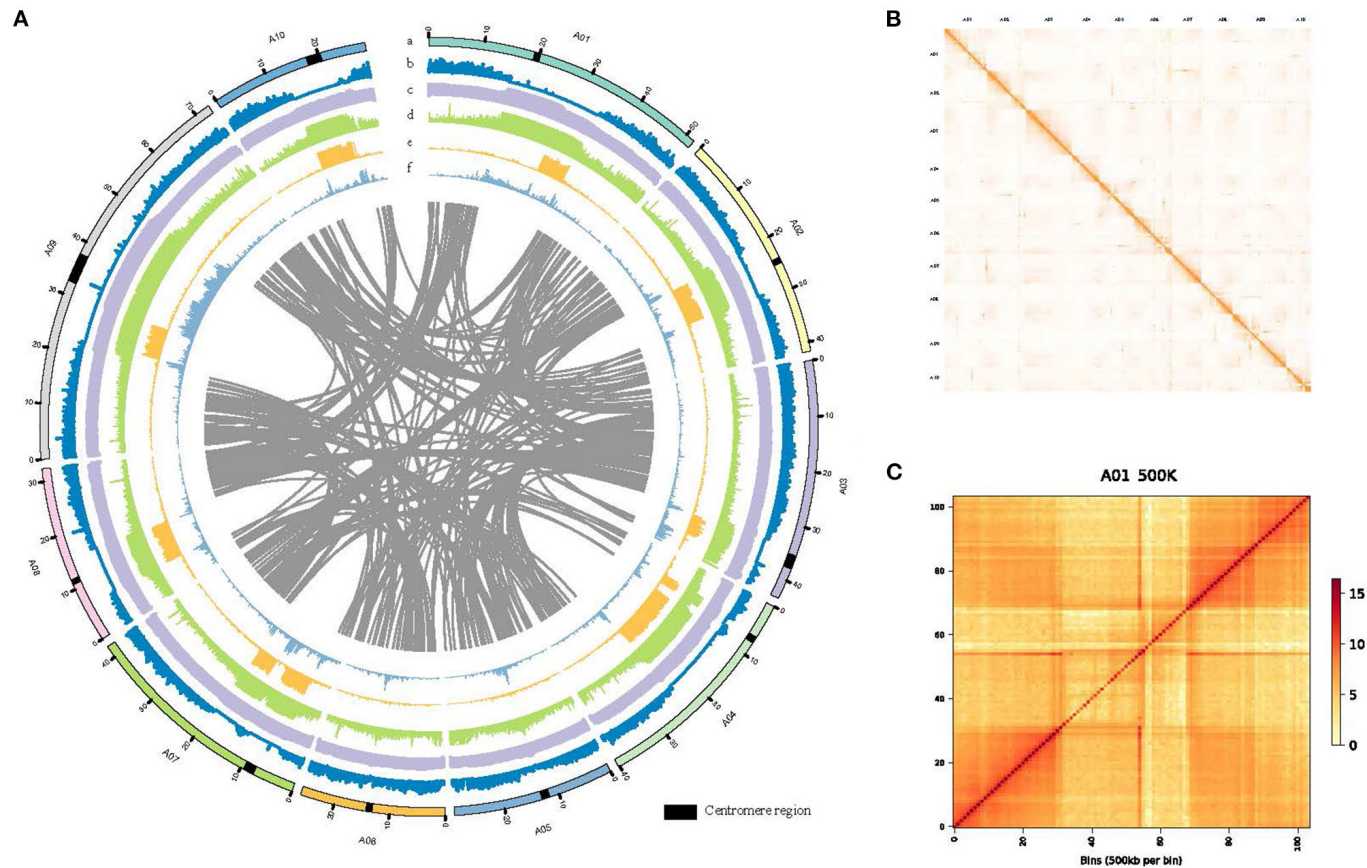
*De novo* genome assembly and annotation of *B.rapa* “Longyou-7” genome. **(A)** Global view of genome features of *B. rapa* “Longyou-7,” Track a shows the 10 pseudochromosomes of *B. rapa* “Longyou-7” denoted by different colors. Track b shows the genome-wide distribution of Gene density. Track c shows the density distribution of GC content. Track d displays the density distribution of transposable elements (TE). Track e shows the density distribution of Copia. Track f shows the density distribution of Gypsy. The red triangles represent the location of the centromeres in all chromosomes; **(B)** Genome-wide Hi-C contact matrix of *B. rapa* “Longyou-7” genome; **(C)** Hi-C map of contact between two 500 kb loci in A01 of *B. rapa* “Longyou-7” genome.

“LY7” and “Lenox” are two winter turnip rape varieties with strong and weak cold tolerance, respectively. Compared to the weak cold-tolerant “Lenox,” “LY7” displayed several distinct character traits such as fewer leaves, smaller and shorter leaves, and longer and larger taproot, which finally leads to its smaller dry leaf weight, larger dry taproot weight, and root/shoot ratio than Lenox (Table 1; Supplementary Figure S1). In addition, LY7 displayed prostrate growth and its shoot apex meristem (SAM) is beneath the ground (Figure 1), which protects it from drastic air temperature change. Furthermore, the leaves of LY7 withered earlier than Lenox, which could reduce water loss and also alleviate further damage transduced from leaves to SAM across winter. In two consecutive years of production experiments in six locations in Gansu province, China, Zhangye, Wuwei, Aolan, Lanzhou, Qingyang, and Jiuquan, the overwintering rate of “Longyou-7” variety of rapeseed reached 90.2% to 97.0% percent, and that of “Lenox” variety rapeseed reached 1.4% to 10.3% percent.

**Table 1:** Statistics of assemblies and annotation in *B. rapa* “Longyou-7” genome.

**Figure**	**Value**
Assembly	
Total contigs	158
Total length (bp)	413,277,474
Min scaffold length (bp)	50,948
Max scaffold length (bp)	72,779,390
Average length (bp)	6,168,320.51
Contig N50 length (bp)	10,226,103
Scaffold N50 length (bp)	42,251,188
(G + C)s (%)	37.05
Annotation	
Repeat length (bp)	223
Repeat ratio (%)	54.35
Total gene number	45,844

### Physiological Characteristics of *B. rapa* “Longyou-7”

When subjected to low temperatures, plants produce increased content of reactive oxygen species (ROS). Excess ROS can result in oxidative damage to cellular membranes and other cellular components, which ultimately leads to cell death. Malondialdehyde (MDA) is a type of ROS generated by the peroxidation of membrane polyunsaturated fatty acids (Esterbauer et al., 1991) and the content of MDA in the plant is often used as a parameter to evaluate the damage to plant cells due to stress. Plant with lower amounts of MDA under low temperature is generally considered more tolerant to cold. To determine whether ROS accumulation is related to the difference in cold tolerance between LY7 and Lenox, we measured the MDA content of plants grown under natural conditions. LY7 and Lenox were sowed in late August and leaves were collected once every month after sowing until December when the temperature went frozen. MDA contents increased in both LY7 and Lenox as the temperature went down from September to December, and the MDA content in Lenox is significantly higher than that in LY7 (Supplementary Figure S2), indicating that cell membranes of LY7 were less damaged by ROS generated by low temperature. Soluble protein has been proven to enhance the cold hardiness of plants, and increasing the content of soluble protein can enhance the cells to retain moisture, improving cold resistance capability (Jung et al., 1967). The content of soluble protein increased from September to October, then decreased, and the content did not show a significant difference between LY7 and Lenox.

ROS-scavenging enzymes play crucial roles in ROS homeostasis, and ascorbate peroxidase (APX), peroxidase (POD), superoxide dismutase (SOD) and catalase (CAT) are four major enzymes that can reduce the accumulation of ROS, weaken the damage to cells and improve the stress resistance of plants (Apel and Hirt, 2004). We measured the activities of these four enzymes and found that the activities of these four enzymes in LY7 are significantly higher than that in Lenox at all time points, suggesting that LY7 could efficiently remove ROS generated by low temperature and protect cells from damage. These physiological indicators may explain the difference in cold tolerance between LY7 and Lenox.

### *De novo* Assembly and Annotation of the “LY7” Genome

To reveal the genetic basis of the super cold tolerance in “LY7,” we sequenced and de novo assembled the genome of “LY7” with high fidelity (HiFi) reads generated by PacBio Circular Consensus Sequencing (CCS) technology. A total of 29 G (equivalent to 68× genome coverage) sequencing data, with 1,767,878 HiFi reads with Q30 of 95.83%, was obtained. The HiFi reads have an average read length of 16,440 bp and read length N50 of 16,344 bp. The maximum read length reached 43,710 bp. We also obtained 64 G high-throughput chromosome conformation capture (Hi-C) reads. Assembly with HiFi reads generated a total of 645 contigs, with a contig N50 size of 10.32 Mb, and a total length of 429 Mb. The longest contig reached 41.70Mb (Table 1). Scaffolding with Hi-C reads resulted in 67 scaffolds with a scaffold N50 size of 42.25 Mb and a total length of 413 Mb. With the assistance of Hi-C data, we anchored 408 Mb to 10 pseudo-chromosomes, with A09 being the longest (72.77 Mb) and A06 being the shortest (26.18 Mb) (Table 1; Figures 1A,B). All the 10 chromosomes show continuous Hi-C signals in the heatmap (Figure 1B), indicating frequent interactions between adjacent loci, and each chromosome can be seen to occupy a separate territory within the nucleus. Strong intrachromosomal interactions are also observed within two chromosome arms, such as A01, where the centromere region can be seen in the Hi-C heatmap, which has a significantly smaller range of interactions than the two chromosome arms (Figure 1C). The GC content of the *B. rapa* genome was 37.05%. A total of 45,844 gene models were annotated. To assess the quality of the genome assembly of “LY7,” we evaluated the completeness of gene models using the single copy embryophyte_odb10 BUSCO dataset which contains 1,614 BUSCO gene sets of core conserved plant genes. Of which, 1,595 genes (98.9%) are intact in the “Longyou-7” genome, 5 genes (0.3%) are fragmented, and 14 genes (0.8%) genes are missed in the “LY7” genome, indicating that the genome assembly has good integrity (Seppey et al., 2019). We also assessed the completeness by mapping the RNA-seq reads to the “LY7” genome and found that more than 92% of HiSeq reads could align properly. These results demonstrated the high completeness of the assembled genome (Supplementary Table S1).

To analyze repetitive sequences, we searched the genome sequence via a combination approach of de novo structure-based analysis and homology-based comparisons referring to previous methods (Schmutz et al., 2010). More than half (54.35%) of genomic sequences were annotated as repeat elements, which is higher than that in the 16 genomes assembled by Cai et al. (2021). The mean length of repeat elements was 232 bp, which is longer than most other samples analyzed in the previous study (Cai et al., 2021). A total of 976,574 transposable elements (TEs) were identified (Supplementary Table S2). As found in other plant genomes, long terminal repeat (LTR)-retrotransposons were the most abundant elements, including 298,537 Copia-like, 33,378 Gypsy-like, and 7,568 unclassified LTR elements, representing 42.5% of all the identified TEs. In addition to class I retrotransposons, 637,091 class II DNA transposons were identified, including 2,496 Tc1/Mariners, 12,784 hATs, 447,564 Mutators, 11,160 PIF/Harbingers and 155,055 Helitrons. These TEs, together with abundant truncated elements and other repetitive fragments, made up 54.81% of the LY7 genome.

The distributions of gene density, GC content, Gypsy, and Copia density on the 10 pseudo-chromosomes are depicted in a circus plot (Figure 1A). Gene density in the two arms was much higher than that in the centromeric regions, which could approximately delimit the locations of the centromeres on chromosomes. The centromere sequences were successfully identified for all chromosomes of LY7 (Figure 1A). The distribution of GC is contrasting to gene density, with the centromeric regions having much higher GC content than the arms. The distributions of transposons along chromosomes are consistent with the GC content, with the density of transposable elements in the centromeric regions much higher than that in the chromosome arms. The two major transposon superfamilies, Gypsy and Copia, are mainly located in the centromere regions, which are also consistent with the GC content. Whereas the distribution of the Copia superfamily transposons was complementary to that of the Gypsy superfamily transposons in the centromere regions (Figure 1A).

### Whole Genome Duplication and Evolution

**Figure 2:**
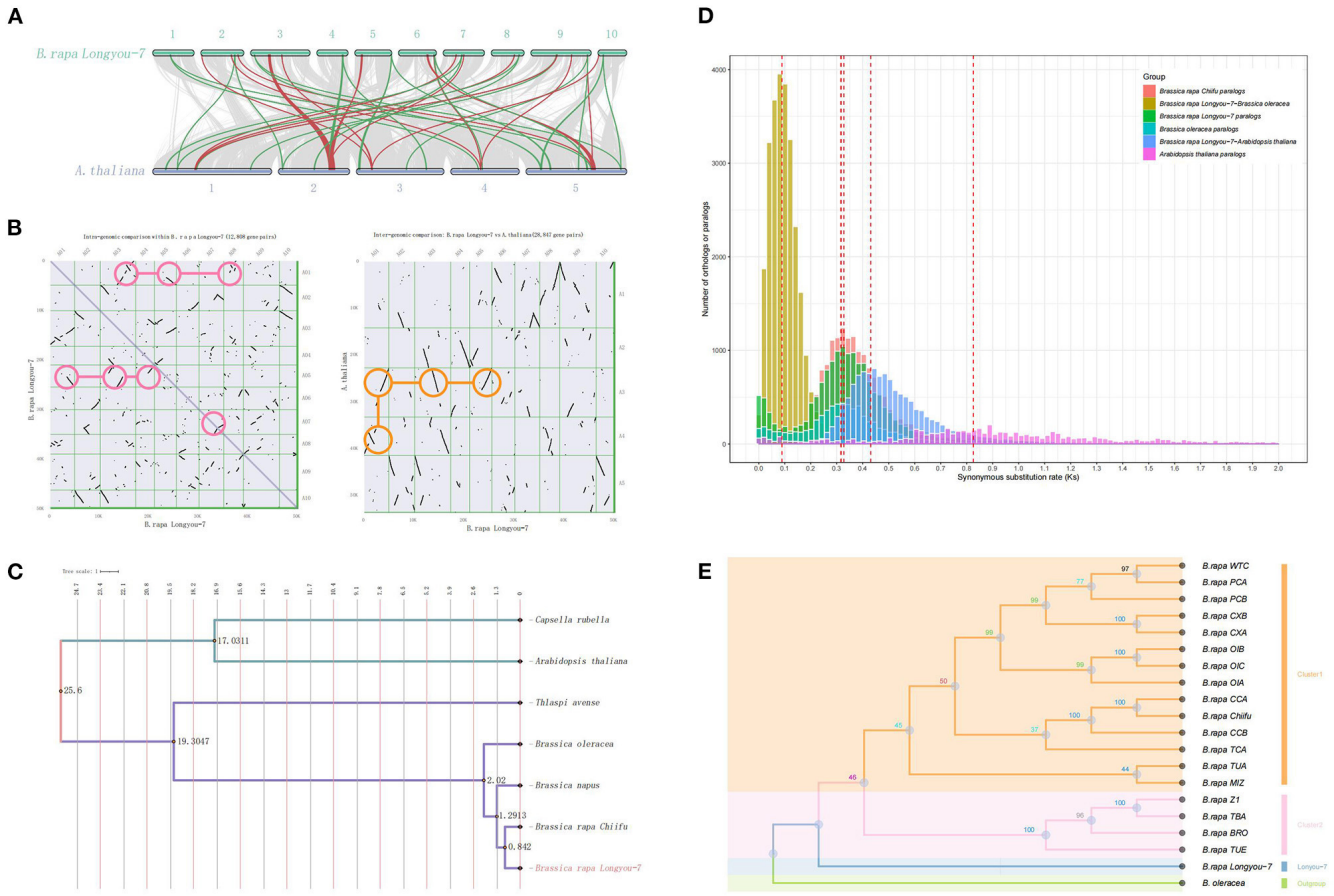
WGT and evolution of *B. rapa* “Longyou-7” genome. **(A)** Macrosynteny patterns reflect a 3:1 syntenic depth between *B. rapa* “Longyou-7” and *A. thaliana* genomes. Green linkages mark examples where three copies of a gene on chromosomes of Arabidopsis are present in the “Longyou-7” genome; **(B)** Dot plot illustrating the comparative analysis within the “Longyou-7” and *Arabidopsis* genomes, the pink circles highlight several major triplication events, the dots represent the synteny gene pairs, and the orange circles highlight an example of 3:1 synteny depth; **(C)** Phylogenetic tree constructed with single-copy orthologs of seven species with divergence time annotated. **(D)** Ks (Synonymous substitution rate) distributions for Brassica rapa paralogs and orthologs with other genomes: *A. thaliana, B. oleracea*, and *B.rapa Chiifu*; **(E)** Species tree constructed using the maximum likelihood method with single-copy ortholog sequences of *B. rapa* “Longyou-7” genome and 18 reported *B. rapa* genomes, with *B. oleracea* as the outgroup.

The *B. rapa* genome has experienced a whole-genome triplication (WGT) event relative to the *A. thaliana* genome (Wang et al., 2011), which was reported to play an important role in the speciation and morphotype diversification of *Brassica* plants (Cheng et al., 2014). Synteny dot plot analysis between the “LY7” genome and the *A. thaliana* genome revealed a 3:1 syntenic depth was identified, which also confirms the extra WGT event reported in the previous study (Wang et al., 2011) that *B. rapa* genome underwent (Figure 2A). Self-alignment analysis revealed long stretches of duplications within the assembled *B. rapa* “Longyou-7” genome among chromosomes, for example, chromosome 1, 3, 5, and 8 (Figure 2B). Intra-chromosomal duplication also exists in *B. rapa* “Longyou-7” genome, which was only found in chromosome 7. Combined with the phylogenetic tree, we can speculate on the occurrence time and species specificity of WGT (Figure 2C). Distribution of synonymous substitutions per synonymous site (Ks) of paralogous genes and syntenic blocks for *B. rapa* “Chiifu” (Wang et al., 2011), *B. rapa* “Longyou-7” and *B. oleracea* (Parkin et al., 2014) confirmed two WGT peaks near Ks = 0.32 and Ks = 0.83, and a divergence peak of *B. rapa* with *A. thaliana* near *Ks* = *0.43*, the synonymous replacement rate is chosen to be 1.5^*^10^−8^ mutations site/year refer to the previous study (Song et al., 2020), so the WGT events in *B. rapa* and *A. thaliana* are estimated to have occurred about 10.7 million years ago and 27.7 million years ago, respectively, according to the formula “T = Ks / 2r.” Phylogenic analysis was performed with some *Brassica ssp*. related species, the WGT event of *B. rapa* was found to happen after the divergence between *Thlaspi avense* and *Brassica ssp*., which is at about 19.3 MYA (Figure 2D). WGD in plants was reported to have a significant contribution to plant adaption (Wu et al., 2020), which also includes adaptation to ambient temperature. The times of the above events were similar to what had been reported (Song et al., 2020; Cai et al., 2021). On the other hand, the phylogenetic tree of *B. rapa* was constructed using the maximum likelihood method using single-copy ortholog sequences among the “Longyou-7” genome and 18 reported *B. rapa* genomes (Cai et al., 2021), with species *B. oleracea* (Parkin et al., 2014) as the outgroup. The results of the clustering showed that the “Longyou-7” genome differed significantly from the other 18 genomes of *B. rapa* and formed a separate branch so that it was necessary to construct the reference genome of LY7 to deepen the understanding of the genetic diversity of *B. rapa*. (Figure 2E).

**Figure 3:**
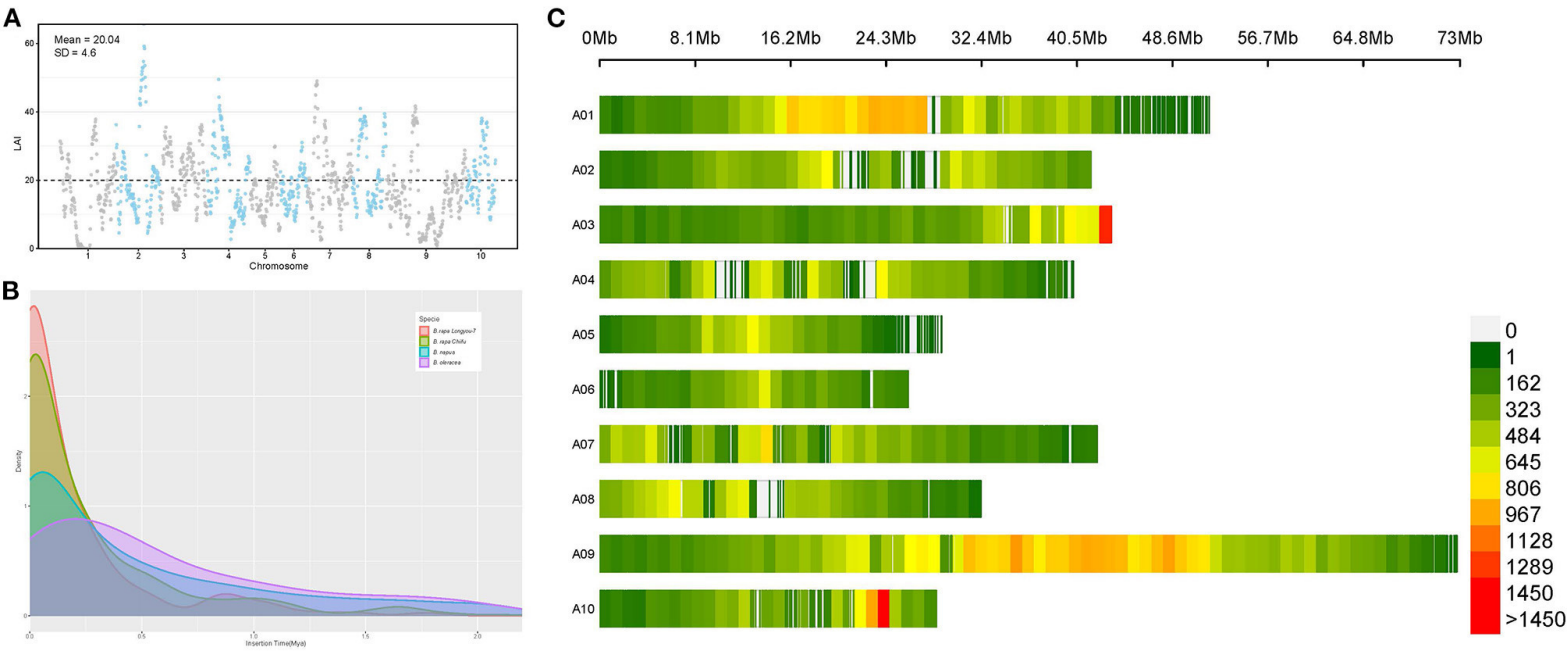
LTR insertion events in *B. rapa* “Longyou-7” genome. **(A)** LAI score reflects the quality of repetitive sequences of *B. rapa* “Longyou-7” genome assembled, with mean LAI = 20.04, SD = 4.6; **(B)** Insertion time distribution of LTR retrotransposons in *B. napus, B. oleracea, B. rapa*, and *B. rapa* “Longyou-7” genomes; **(C)** The distribution of LTR-RTs density in each chromosome of *B. rapa* “Longyou-7”.

The abundance of repetitive sequences is thought to be the main challenge of plant genome assembly and long terminal repeat retrotransposons (LTR-RTs) are the dominant repetitive sequences that are poorly assembled in draft genomes. LTR Assembly Index (LAI) is reported to be used for evaluating assembly continuity using LTR-RTs (Ou et al., 2018). Here we calculated the LAI of 10 chromosomes of our *B. rapa* genome. Our research performs an LAI estimate toward the assembled *B. rapa* genome to visualize its assembly quality. LAI score reflexes the assembly quality of repetitive region sequences. The mean LAI score of our genome is 20.04 with a standard deviation is 4.6, which suggests the assembly of our *B. rapa* genome shows high quality. However, some chromosome regions also present low quality (Figure 3A). For example, some regions of chromosome A09 are found with LAI scores under 10, which means the assembly of this region might be implausible (Ou et al., 2018). The assembly results of the complete repetitive elements provided us with an opportunity to accurately analyze the LTR-RTs insertion burst events in the *B. rapa* genome, which are considered to be the driving force of WGT. To investigate the evolutionary dynamics of LTR-RTs, the insertion time in *B. rapa* “Longyou-7,” *B. rapa* “Chiifu,” *B. oleracea* (Parkin et al., 2014), and *B. napus* was estimated (Figure 3B). The density plot indicated that the *B. rapa* “Longyou-7” genome has a comparatively highest proportion of recent insertions, which is considered to contribute to the larger genome size (413.27Mb) than *B. rapa* “Chiifu” (353.14Mb) (Wang et al., 2011). The LTR-RTs density analysis shows the general existence of LTR-RTs insertion event within all the chromosomes in *B. rapa* with two blocks presents extra high LTR-RTs density, at the end of chromosome A03 and the site about 24 Mb on chromosome 10 (Figure 3C). Large scale block with high LTR-RT density was also discovered in chromosome 1 and chromosome 9 at the 16Mb-28Mb and 30Mb-50Mb regions respectively (Figure 3C). Another peak in LTR-RTs insertion density occurred about 1 million years ago and can be seen within both the “Chiifu” and “Longyou-7” genomes, which may suggest an ancient LTR insertion event. The activity of transposable elements (TEs) including LTR-RTs is thought to cause various genetic diversities, including the adaption of the environment (Wu et al., 2020).

### Graph-Based Pan-Genome

**Figure 4:**
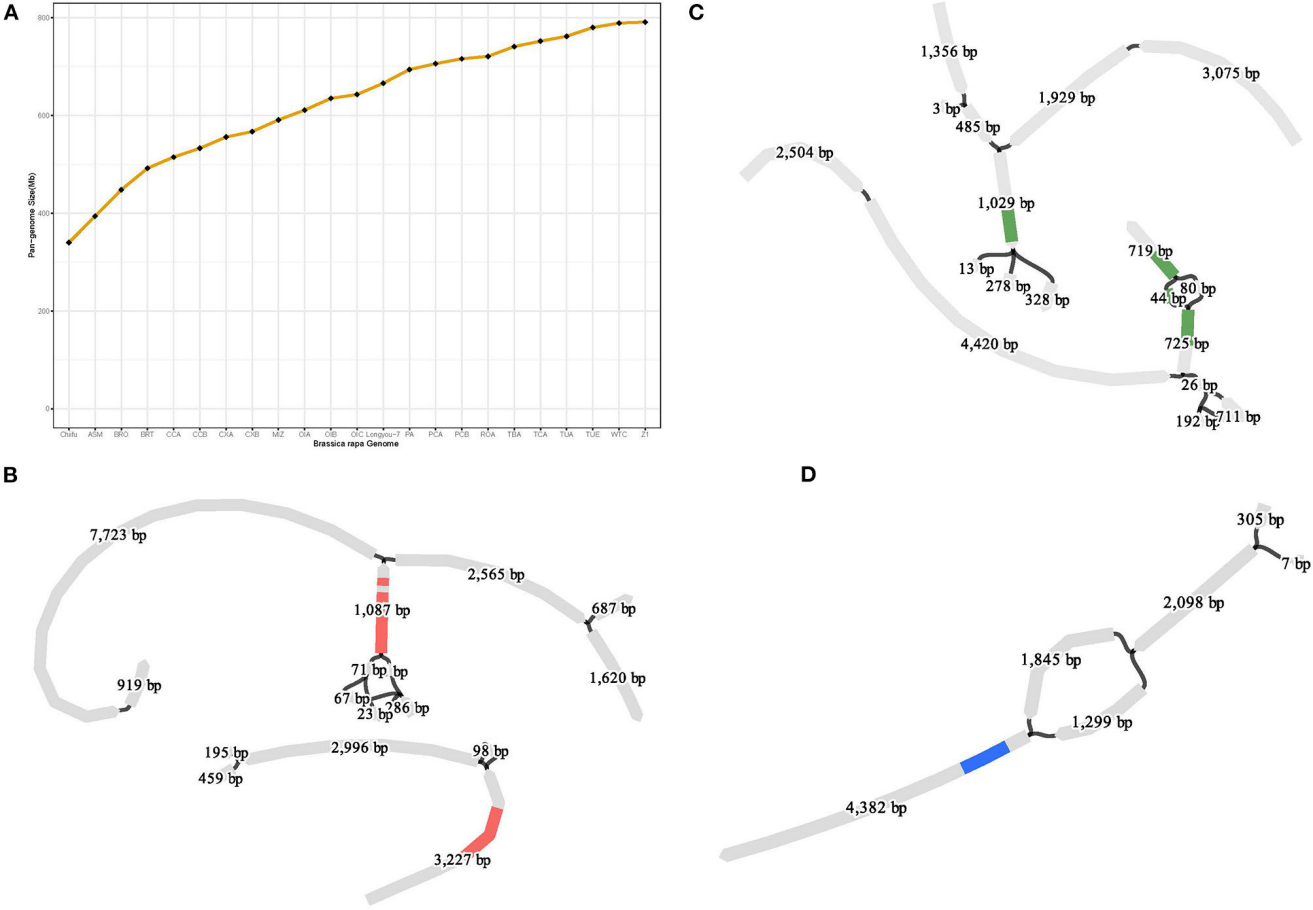
Graph-based pan-genome constructed with 23 *B. rapa* genomes currently exist. **(A)** Pan-genome size increased with more genomes were used; **(B)** Two copies of gene *TCP4* were detected in pan-genome and the SVs around them; **(C)** Two copies of the gene *ERD10* detected in the pan-genome we constructed and the SVs located inside one of the copies **(D)** Structural variants detected in the pan-genome upstream of the gene *ERF105*.

Compared with the reference genome (Chiifu), the genome of LY7 contains 2,174,693 SNPs and 580,073 Indels, involving 4,381,775 bp sequences. But this is far from representing the genetic diversity of species in *B. rapa*. A pan-genome represents an approximation of the entire gene repertoire and provides an important resource for the identification of genetic variants, particularly for larger structural variants such as presence/absence variants (PAVs) and copy number variants (CNVs) of a species. A graph-based pan-genome uses substitutable sequences in a population to represent the variants present at each locus (Li et al., 2022), which can be visualizable and facilitate fast and accurate identification and genotyping of larger SVs within and close to specific genes. To capture the entire genomic diversity, we constructed a high-resolution graph-based pan-genome of *B. rapa* using the assembled genome of “LY7” together with 22 previously reported *B. rapa* genomes representing different morphotypes and ecotypes (Cai et al., 2021) (Supplementary Table S3). The *B. rapa* genome of “Chiifu” was used as the reference genome and the other 22 genomes including LY7 were iteratively aligned to the genome of “Chiifu” using Minigraph (Li et al., 2020) (Supplementary Table S3). A graphical pan-genome that contained a total of 91,308 structural variations was finally obtained. The size of the pan-genome gradually increased as the number of genomes increased, and eventually leveled off at about 800Mb, indicating that the pan-genome is close to saturation (Figure 4A). The graphical pan-genome contains 642,697 sequence fragments, of which 172,811 are from the reference genome, and the rests are PAV identified from other 22 *B. rapa* genomes.

### SVs Related to Cold Tolerance

Cold regulated (COR) genes have been isolated and identified from *Arabidopsis thaliana*, canola, rice, and other plants (Hajela et al., 1990; Thomashow et al., 1997; Park et al., 2015). The COR genes encode various functional proteins to resist cold stress and improve cold resistance, including *DREB, CBF, NAC, MYB, bZIP*, and *WRKY* (Jaglo-Ottosen et al., 1998; Yoo et al., 2007; Liao et al., 2008, p. 62; Kim et al., 2016; Liu et al., 2019). To reveal the genetic basis of the strong cold tolerance of LY7, we collected a total of 97 COR genes from literature, including 29 genes from Arabidopsis, 13 genes from rice, three genes from *B. rapa*, and 53 genes from *B. napus* (Supplementary Table S1). BLAST analysis using amino acid sequences of these genes as query identified 53 orthologous genes in the LY7 genome (Supplementary Table S1). Of the 53 COR genes, 40 were found to be multiple copy genes (Supplementary Tables S4, S5), which may be related to the WGT event mentioned earlier. Compared to the reference genome of Chiifu, we identified copy number variations (CNV) in 39 out of the 40 multiple copy genes. In addition, with the assistance of the graph-based pan-genome, we found SVs within or upstream (<2 kb) of 17 out of the 40 COR genes. For example, *TCP4* in Arabidopsis encodes a transcription factor that coordinates growth processes during leaf development (Martín-Trillo and Cubas, 2010), which is mediated by miR319. miR319 and its target *TCP4* can act as switches that turn on secondary cell wall synthesis, which is reported to be associated with cold tolerance in plants (Zeng et al., 2018). *Bra032365* is an orthologous gene of *TCP4* in *B. rapa*. It has only one copy in Chiifu, but three copies in LY7 (Supplementary Table S5). BLAST search in the graph-based pan-genome identified CNVs and SVs of *Bra032365* in all the 23 *B. rapa* genomes (Figure 4B). *Early Responsive to Dehydration 10* (*ERD10*) is reported to play role in the protection of the plants from various stresses, including cold and dehydration (Kim and Nam, 2010). Two orthologous copies of *ERD10, Bra012230*, and *Bra025819* located on different chromosomes, were identified in Chiifu, while three copies were identified in LY7. In addition, SVs were identified in both *Bra012230* and *Bra025819* (Figure 4C). The *ethylene response factor* (*ERF*) gene family encodes plant-specific transcription factors. *Bra035732* is an orthologous gene of *ERF105*, which was reported to play role in freezing tolerance and cold acclimation of *A. thaliana* (Bolt et al., 2017). Large SVs were detected upstream of *Bra035732* (Figure 4D). The CNVs and SVs identified in these COR genes may be associated with cold tolerance in *B. rapa*.

### The Potential Impact of SVs Related to Cold Tolerance

**Figure 5:**
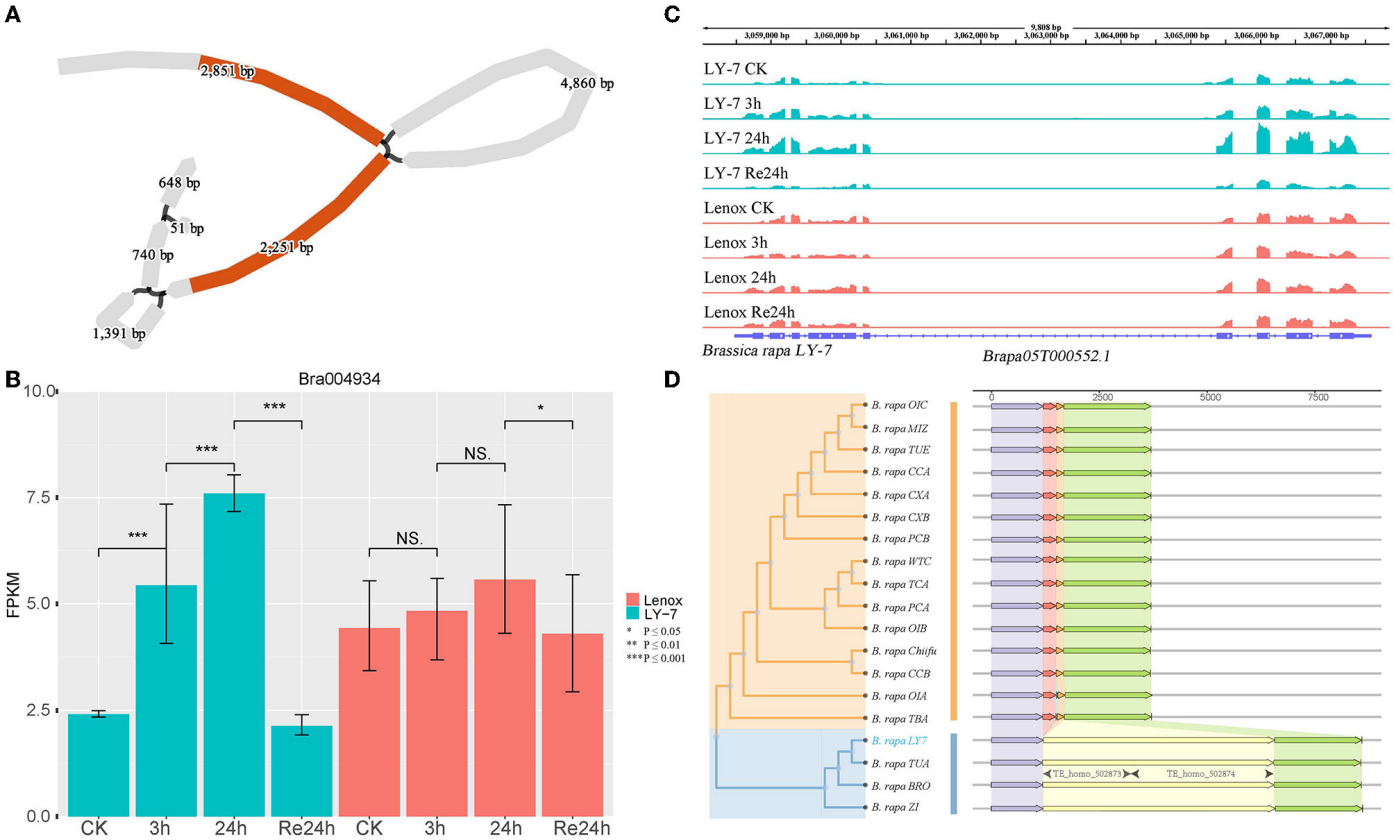
Structure variant of *HDG1* in *B. rapa* “Longyou-7” genome. **(A)** BLAST hit of gene *HDG1* in the pan-genome and 4860bp of structural variation detected within it; **(B)** The differential expression of *HDG1* between “Longyou-7” and “Lenox” within 48 h; **(C)** Coverage of RNAseq reads in the region of gene *HDG1* in “Longyou-7” genome and their abundance; **(D)** Neighbor-joining tree clustering and the gene structure characteristics of gene *HDG1*.

To further reveal if the CNVs and SVs identified in the 40 COR genes are associated with cold tolerance, we analyzed the differences in expression levels of these genes before and after cold treatment. Plants of LY7 and Lenox grow in a 22 °C growth chamber (with 16/8h light/dark cycle) to a six-leaf stage and then were transferred to a 4 °C growth chamber for 3 and 24 h, and then recovered at 22 °C for 24h. Growth points of these plants were collected for RNA-seq (Ma et al., 2019). Of the 17 genes having structural variations within coding sequence regions (CDS) or upstream regulatory sequences, two genes, *HDG1* and *BrANS3*, displayed different expression patterns between LY7 and Lenox, while the other genes showed similar expression patterns in LY7 and Lenox before and after cold treatment. *HDG1*, encoding a protein in the homeodomain (HD)-START transcription factor family also known as the Class IV Homeodomain-Leucine Zipper transcription factor family (Nakamura et al., 2006), was previously reported to assist with the growth and development of plants under unfavorable environments including cold stress (Sharif et al., 2021). Overexpressing *HDG1* in plants significantly influenced the root system and improved the resistance to stresses (Horstman et al., 2015). *Bra004934* is a homologous gene of *HDG1*. With the graph-based pan-genome, we identified a 4860 bp LTR inserted in the first intron of *HDG1* in “LY7” (Figure 5A). Alignment of RNA-seq reads to the LY7 genome indicated that the insertion of LTR in the first intron did not change the protein-coding sequence and disrupted the transcription of *HDG1* (Figure 5C). Further comparative analysis identified the same LTR in *HDG1* of BRO (Broccolieto), TUA (Turnip), and Z1 (sarson type) (Cai et al., 2021) genomes (Figure 5D). The expression level of *HDG1* in “Longyou-7” increased 2 folds after 3h cold treatment and four folds after 24h cold treatment (*P* = 0.01). Its expression level was restored to the untreated level after recovery at 22°C for 24h (Figures 5B,C). Whereas the expression level of *HDG1* in Lenox did not show a significant difference before and after cold treatment (Figures 5B,C). Thus, the differential expression of *HDG1* in “LY7” is thought to be associated with the cold-tolerance, and the LTR inserted in the first intron of *HDG1* might be cold-inducible. The structural variation in *HDG1* was further examined in 80 samples from the resequencing dataset of spring Chinese cabbage populations reported in previous studies (Su et al., 2018), and the results showed that the structural variation was detected in 82.5% of the samples, indicating that the structural variation is widespread in cold-tolerant spring cabbage varieties (Supplementary Table S6).

**Figure 6:**
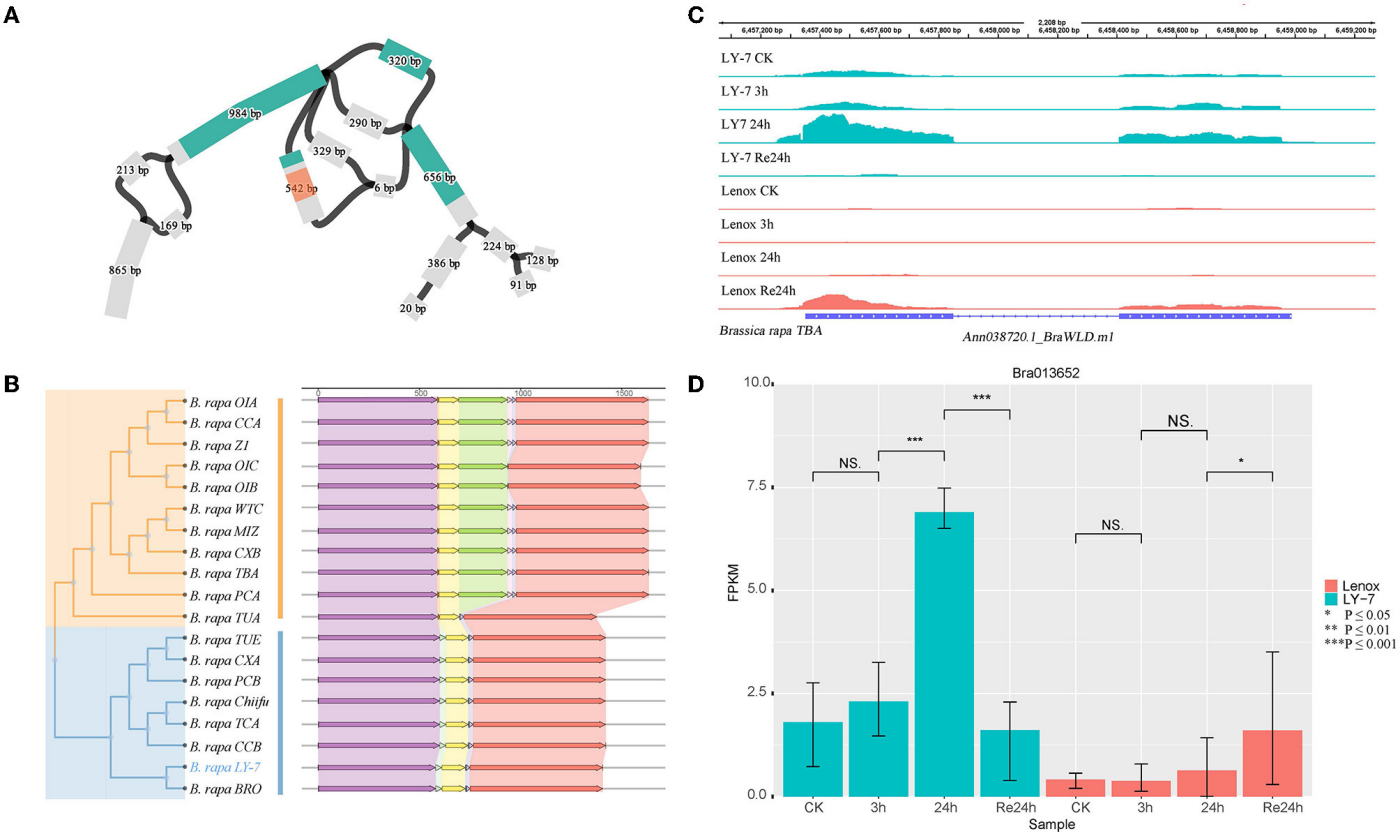
Structure variant of *BrANS3* in *B. rapa* “Longyou-7” genome. **(A)** BLAST hit of *BrANS3* gene of “Longyou-7” genome (blue) and a 240 bp of structural variation (red transparent) detected within the graph-based pan-genome; **(B)** Neighbor-joining tree clustering and the gene structure characteristics of *BrANS3* gene; **(C)** Coverage of RNA-Seq reads in the region of *BrANS3* gene in “TBA” (From Tibet, China) genome and their abundance; **(D)** The differential expression of *BrANS3* between “Longyou-7” and “Lenox” cultivars within 48 h.

Anthocyanins are water-soluble flavonoid pigments widely distributed in the petals, fruits, stems, and leaves of plants (Potapovich and Kostyuk, 2003; Lo Piero et al., 2005; Yuan et al., 2009). In addition, anthocyanins are natural antioxidants that can strongly scavenge free radicals and reactive oxygen species (ROS) (Gould, 2004). Anthocyanidin synthase (ANS) is one of the structural genes encoding multiple enzymes in anthocyanin biosynthesis. *BrANS3* (*Bra013652*) was reported to be strongly associated with anthocyanin accumulation and resistance to cold stress of *B. rapa* (Ahmed et al., 2015). In the constructed graph-based pangenome, we detected structural variations within this gene (Figure 6A). A *Mariner* insertion of about 240 bp was identified in 10 genomes including OIA, CCA, Z1, OIC, OIB, WTC, MIZ, CXB, TBA, and PCA, but not in the other genomes including the two-known cold-tolerant varieties “LY7” and “Chiifu” (Figure 6B). Due to the SNPs in this structural variation, four branches representing four different allelic genotypes can be observed in the graph-based pan-genome (Figure 6A). *BrANS3* was differentially expressed between “LY7” and “Lenox” varieties before cold treatment. The expression level of *BrANS3* was very low in Lenox before cold treatment and not elevated after cold treatment, while the expression level in LY7 was significantly increased after 24h cold treatment (Figure 6C). We further demonstrated that this gene was differentially expressed in cold-tolerant and cold-sensitive varieties by using the genome of the “TBA” variety, which has the *Mariner* insertion of ~240 bp in *BrANS3*, as the reference genome for transcriptome mapping, and found that the insertion did not change the protein-coding sequence of this gene (Figures 6C,D). Therefore, we speculate that the insertion of this fragment may be widely present inside the *BrANS3* of cold-sensitive rapeseed varieties, hindering the expression of the gene and thus causing rapeseed to exhibit cold-sensitive traits, while *BrANS3* without the insertion can express normally and improve the cold tolerance of rapeseed. Alignment of the 80 resequencing data mentioned earlier (Su et al., 2018) to the TBA variety genome revealed that the structural variant was not widespread in the spring Chinese cabbage population and was detected in only 12.5% of the samples (Supplementary Table S7). A weighted gene co-expression network analysis (WGCNA) was performed on the transcriptome data (Ma et al., 2019), and the top 100 co-expressed genes with weight values were screened, five of which contained cold response terms in the GO annotation information (Ma et al., 2019). Their interactions were predicted in the STRING database, and from the results, three of them are located upstream of gene *BrANS3* and may play a regulatory role in the expression of *BrANS3*.

## Discussion

In this study, we sequenced and assembled for the first time a high-quality chromosome-level genome of rapeseed cold-tolerant variety “Longyou-7” and performed a series of correlation analyses based on the assembled genome to elucidate cold-tolerance traits in this cultivar. Comparative genomic analysis of the “Longyou-7” genome with some other cruciferous plant genomes identified the whole-genome triplication event of *B. rapa* in the “Longyou-7” genome. Analysis of TEs revealed a recent LTR insertion event in the “Longyou-7” genome, which is thought to be possibly related to cold adaption in this variety of rapeseed. Together with the currently existing 22 *B. rapa* genomes, we build a graph-based pan-genome with high resolution. Subsequently, we collected 97 genes (Supplementary Table S1) that have been reported to be associated with cold stress located in rice, kale, oilseed rape, and Arabidopsis, and extracted their homologs in rapeseed based on the synteny relationship between these genomes of these species and rapeseed genomes, and further looked for SVs within these genes with the constructed pan-genomes. The transcriptome data of “Longyou-7” reported in previous studies with Lenox (Ma et al., 2019) were used to see the expression of homologous genes with structural variants. We detected SVs in 16 cold tolerance-related homologs (Supplementary Table S4) in the “Longyou-7” genome and two of them, *HDG1* and *BrANS3*, were differentially expressed before and after cold treatment between “Longou-7” and “Lenox” varieties. Among them, *HDG1* was previously reported to enhance plant tolerance to cold stress (Horstman et al., 2015), and in this study, we detected a 4,860 bp structural variation in the internal intron region of this gene in the cold-tolerant “Longyou-7” variety, which was further confirmed to be due to the insertion of two Copia LTR retrotransposons. *BrANS3* was previously reported to be strongly associated with cold tolerance in *B. rapa* (Ahmed et al., 2015), and here a deletion of about 240 bp was detected in the internal intron region of this gene within both known cold-tolerant species, “Longyou-7” and “Chiifu.” We further supported these two findings by sequence-structure analysis. Transcript coverage showed that the structural variation in both genes did not affect their protein-coding sequences, and differential expression of both genes was detected between cold-tolerant and cold-sensitive varieties. Therefore, the structural variants detected in the cold-tolerance-related genes *HDG1* and *BrANS3* are thought to have led to their differential expression between cold-tolerant and cold-sensitive varieties, and consequently to the cold-tolerant phenotype exhibited by the “Longyou-7” variety.

Collectively, our research provides a high-quality genomic resource for the study of the cold-tolerant phenotype in *B. rapa*. Results of the comparative genomic analysis confirmed the WGT event experienced by *B. rapa* “Longyou-7” genome. Moreover, the constructed graph-based pan-genome could serve as a resource for the excavation of cold tolerance-related SVs of *B. rapa*. Furthermore, we uncovered two possible SVs that contribute to the cold-tolerant trait of *B. rapa* variant “Longyou-7,” which reveals new insights into the cold tolerance of rapeseed.

## Materials and Methods

### Plant Materials

“Longyou-7” is a winter turnip rape with super freeze-tolerance bred by scientists at Gansu Agricultural University (Lanzhou, China). They crossed a landrace with strong cold resistance, Chenjiazui winter turnip rape, with high-yielding Tianyou 4 with weak cold resistance in 1996 (Sun et al., 2011). The F2, F3, and higher generations family lines were grown at multiple locations around Lanzhou city (latitude: 34°33′-39°46′N; longitude: 103°.82′E; Altitude:1,083–1,477 m,) under natural conditions in winter. Families with overwinter survival rate higher than 80% were selected for each generation. One of the family lines with super cold resistance was registered as “Longyou-7” in 2007, which can survive in the winter in the region between N34° and N48° and under as low as−31.9 °C environment with a survival rate higher than 80% (Zhou et al., 2014). Lenox is a freeze-sensitive variety also bred by scientists at Gansu Agricultural University. It has a overwinter survival rate of 0-10% at Tianshui (34°36′ N, 105°39′ E, the annual average temperature is 12.9°C), Huining (35°89′ N, 104°62′ E, the annual average temperature is 9.2°C) and Qingyang (35°38′ N, 107°35′ E, the annual average temperature is 10°C).

We also measured the activity of other ROS scavenging enzymes, including superoxide dismutase (SOD), catalase (CAT), glutathione reductase (GR), and peroxidase, in the ospp18 mutant and wild-type plants. Among these ROS scavenging enzymes, the activity of GR was significantly lower in the ospp18 mutant than in the wild type under both the drought stress and non-stress conditions (Figure 6B).

### Measurements of Phenotypic and Physiological Traits

Longyou 7 and Lenox were sowed at the experimental farm of Gansu Agricultural University, Yongdeng County, Gansu, on August 20, 2020. Field management was conducted as regular agricultural practice. The rapeseed leaves and roots were collected when growth reached the five-leaf stage (October).

Leaves were collected every 1 month after sowing from plants grown in the field until December. Root shoot ratio refers to the ratio of fresh weight or dry weight of the underground part and aboveground part of rapeseed. Oxidative damage was estimated by measuring the content of MDA as described previously (Du et al., 2010). The content of soluble protein (SP) was measured as previously described (Arminian and Dehghani Bidgoli, 2019). The activity of ROS scavenging enzymes, such as ascorbate peroxidase (APX) activity, peroxidase (POD) activity (Quiroga et al., 2000), superoxide dismutase (SOD) activity, catalase (CAT) activity, were measured as described in previous studies (Quiroga et al., 2000; Yao et al., 2011; Zhang et al., 2013).

### Library Construction and Sequencing

Genomic DNA was extracted following the method by which genomic DNA was extracted for Illumina and Pacbio library construction and sequencing. Libraries for Pacbio-HiFi sequencing were constructed using the SMRTbell Express Template Prep Kit v2.0 following the manufacturer”s protocols provided by PacBio Company and sequenced on the PacBio platform. The created Hi-C library was digested into units with Dpn II and sequenced by Illumina HiSeq 4000 platform with 150 bp reads length with a 200 bp insert size and sequenced on Illumina platform. Library for Illumina pair-end genome sequencing was built following the provided standard protocol.

### Genome Assembly

The *B. rapa* “Longyou-7” genome was *de novo* assembled using the PacBio SMRT data. Falcon was used for performing subreads polishing and contigs assembly (falcon−201711.02−16.04 -py 2.7), and parameter length_cut_off_pr was set at 6,000. Canu v1.6 (Koren et al., 2017) was used for the assembly of subreads polished by Falcon (Chin et al., 2016), and parameter correctedErrorRate was set at 0.05. Then the PacBio reads were mapped to the draft contigs by pbalign, whose result was polished with Quiver (Chin et al., 2013) using the arrow algorithm. The gained contigs were polished with Illumina PE reads (insertion size 350bp) by pilon1.18. Unique sequences generated by Canu were not found in Falcon assembly. Hi-C reads were used for scaffolding the draft assembly genome using the 3D-DNA pipeline (Dudchenko et al., 2017), with the parameter -i set as 1 and -r set as 5. Hi-C reads were aligned to the polished contigs following the reported Juicer pipeline (Durand et al., 2016b). The result of 3D-DNA was polished using Juicebox (Durand et al., 2016a). We finally got 10 chromosome-length scaffolds. The completeness and accuracy of *B. rapa* 'Longyou-7' assembly were assessed using BUSCO (Seppey et al., 2019) with the embryophyte_odb10 dataset.

### Repeat Element Annotation

An extensive *de-novo* TE Annotator (EDTA) pipeline (Ou et al., 2019) was used for building a whole-genome *de novo* repeat library and performing the identification of the transposable elements (TEs). The *de novo* detection of long terminal repeat (LTR) retrotransposons in *B. rapa* “Longyou-7” genome we detected was performed with LTR_Finder (Xu and Wang, 2007) and LTRharvest (Ellinghaus et al., 2008), the results of which were further filtered with LTR_retriever (Ou and Jiang, 2018), and the LTR insertion time was calculated at the same time. The extracted LTRs were used for the calculating LTR Assembly Index (LAI) to evaluate the assembly continuity of *B. rapa* “Longyou-7” genome using LTR-RTs by LAI program (Ou et al., 2018).

### Gene Prediction and Annotation

Multiple strategies were used for the prediction of gene structure including homologous prediction, *de novo* prediction, and evidence-based prediction. *De novo* prediction software including Augustus (Stanke et al., 2006), GlimmerHMM (Majoros et al., 2004), and SNAP (Söllner et al., 1993). Genewise (Birney et al., 2004) was used for the homologous prediction of gene structure. RNA-Seq data of mixed tissue were used for evidence-based prediction by EVidenceModeler (Haas et al., 2008). The correction and addition of UTR, variable clipping, and other information were performed with PASA (Haas et al., 2008).

### Whole Genome Triplication in *B. rapa* “Longyou-7” Genome

To study the evolution of *B. rapa* “Longyou-7” genome, syntenic blocks between *B. rapa* “Longyou-7” and *A. thaliana* were defined and presented by the Mcscan python version (Tang et al., 2008) with default parameters. The analysis revealed long stretches of triplication within the *B. rapa* “Longyou-7” genome assembled, and they were found to be not only inter-chromosomal but also intra-chromosomal. Paralog analysis within *B. rapa* “Longyou-7” genome was performed with reciprocal best hits (RBH) from self-BLASTp using all the primary protein sequences. Self-BLASTp was performed using python script blast_rph.py (https://github.com/peterjc/galaxy_blast/blob/master/tools/blast_rbh/blast_rbh.py) in the galaxy_blast package (Cock et al., 2015). Further analysis of the synonymous substitution rate (Ks) of RBH gene pairs was calculated based on the MA model by KaKs_Calculator v2.0 (Wang et al., 2010) and ParaAT2.0 (Zhang et al., 2012) to confirm the WGT event *B. rapa* “Longyou-7” genome underwent and the time it happened and 22,078 RHB paralogous gene pairs in the *B. rapa* genome. Both the WGT peak in *B. rapa* “Longyou-7” genome and *B. rapa* current reference genome and the peak indicates the divergence between *B. rapa* and other *Brassica ssp*. was detected. Single copy ortholog protein sequences among *A. thaliana* (Sloan et al., 2018), *Capsella rubella* (Slotte et al., 2013), *Thlaspi arvense* (Dorn et al., 2015), *B. oleracea* (Parkin et al., 2014), *B. napus* “ZS11” (Song et al., 2020), *B. rapa* “Chiifu” (Wang et al., 2011) and *B. rapa* “Longyou-7” was generated with OrthoFinder (Emms and Kelly, 2019), and then aligned with MUSCLE (Edgar, 2004) based on the alignment result phylogenetic tree was constructed using RAxML (Stamatakis, 2014) and r8s (Sanderson, 2003) was used for estimating of the split times. The species phylogenic tree of the 18 reported *B. rapa* genomes (Cai et al., 2021) and our “Longyou-7” assembly was constructed using the same process, with species *B. oleracea* being used as the outgroup. R package “ggtree” was used for phylogenetic tree mapping and landscaping.

### RNA-seq Data Analysis

RNAseq data set was obtained from the published research (Ma et al., 2019) (NCBI accession number: SRP179662). A quality check was performed with FastQC. Trimmomatic (Bolger et al., 2014) was used for filtering reads with low quality to gain clean reads. Clean reads were aligned to *B. rapa* reference genome (Wang et al., 2011) using STAR (Dobin et al., 2013), and samtools was used to transform SAM file into BAM file. The count of reads mapped to the reference genome was counted using RSEM (Li and Dewey, 2011). Cufflinks (Trapnell et al., 2010) was used to calculate fragments Per Kilobase of exon model per Million mapped fragments (FPKM). Differential expression analysis was performed with “DESeq2.” The statistical analysis and mapping of expression levels of gene *HDG1* and *BrANS3* were performed using the R packages “ggplot2” and “ggsignif,” and the comparison of reads on the reference genome was further viewed and analyzed using igvtools (v2.11.2).

### Graph-Based Pan-Genome Construction

Twenty-two previously reported *B. rapa* genomes were downloaded from the Brassicaceae Database (BRAD) (http://www.brassicadb.cn/) and National Center for Biotechnology Information (NCBI) (https://www.ncbi.nlm.nih.gov/) (Belser et al., 2018; Zhang et al., 2018; Cai et al., 2021; Li et al., 2021), including heading Chinese Cabbage, turnips (Chinese and European turnips), sarsons (sarson, rapid cycling, and oilseed), pak choi (pak choi, wutacai, and caixin), and Japanese morphotype (mizuna). Of these genomes, the annotation information of four genomes (ASM, BRT, PA, ROA) is not available. The 22 previously reported *B. rapa* genomes and the *B. rapa* “Longyou-7” genome were built into a variant graph using minigraph following the published process with default parameters (Li et al., 2020). The genome of variety “Chiifu” (Wang et al., 2011) was used as the reference genome, and the genomes of the other 22 varieties were mapped iteratively, one by one, using in-house shell scripts, for sequence-to-graph mapping, and the generated GFA format file was used to call bubbles, which represent structural variants, using gfatools (v0.4-r214-dirty) (https://github.com/lh3/gfatools). The constructed graph-based pan-genome in GFA format was visualized using Bandage (v0.8.1) (Wick et al., 2015).

### Identification of SVs Related to Cold Tolerance

To find the SVs related to the cold tolerance phenotype of *B. rapa* “Longyou-7”, 97 cold tolerance related genes from *B. rapa, B. oleracea, A. thaliana*, and rice (*Oryza sativa*) (Supplementary Table S1) was collected and aligned to19 genomes including 18 *B. rapa* genomes reported in the previous study (Cai et al., 2021) and “Longyou-7” genome with Mcscan python version (Tang et al., 2008). Homologous genes identified in these *B. rapa* genomes were aligned to the graph-based pan-genome to check if there are SVs within or around them. Here we selected *HDG1* (gene ID: *Bra004934*) and *BrANS3* (gene ID: *Bra013652*) to perform further checks. The longest transcript of it was extracted according to the GFF file with an in-house bash script. The inter-genome genes relationship was built with the MCScan python version (Tang et al., 2008). Sequences of *HDG1* homologous genes in the 18 *B. rapa* genome were extracted with in-house scripts. The synteny blocks were analyzed using Mauve (v2015-02-26) (Darling et al., 2004) and presented with the R package “gggenes.” The neighbor-joining tree of sequences of each gene was constructed with MEGA11 (Tamura et al., 2021) with default parameters and drawn using the R package “ggtree.” Information on transposable elements located in the *HDG1* of the “Longyou-7” genome was obtained from the output of the EDTA pipeline (Ou et al., 2019).

### Gene Coexpression Network

Gene co-expression network analysis was performed using transcriptomic data of 41,019 genes with the R package “WGCNA” (Langfelder and Horvath, 2008). Terrain Overlap Matrix (TOM) was calculated with the “blockwiseModules” function in the WGCNA package, where the TOMType was set to “unsigned,” and the minimum module size was set to 30. The co-expression network was constructed by a one-step method, maxBlockSize was set to 50000. From the results, all genes interacting with the *BrANS3* were extracted, ranked by weight value, and the top 100 were selected for further analysis, and the interactions between the proteins encoded by these genes were predicted using the STRING database (https://string-db.org) (Szklarczyk et al., 2021). The visualization of the interaction network was implemented using Cytoscape (v3.9.0) (Shannon et al., 2003).

### Alignment of Resequencing Data

Eighty population resequencing data were obtained from the dataset of previous studies and matched to the corresponding reference genomes using BWA (v0.7.17-r1188) (Li, 2013). The alignment results were converted to BAM format files using samtools (v1.9) (Li et al., 2009) and sorted. Finally, the sorted BAM files were counted using bedtools (v2.30.0) (Quinlan and Hall, 2010).

## Data Availability Statement

The original contributions presented in the study are publicly available. This data can be found here: NCBI, SRR18959686.

## Author Contributions

JW and WS conceived and designed the study. X-DX analyzed the data. LL, LM, YP, WW, X-YH, J-MS, GL, YF, and XL contributed materials and analysis tools. JW and X-DX wrote the paper. KL revised the manuscript. All authors contributed to the article and approved the submitted version.

## Funding

The study was funded by the Research Program Sponsored by State Key Laboratory of Aridland Crop Science, Gansu Agricultural University (No. GSCS-2020-Z1) and the China Agriculture Research System of MOF and MARA (CARS-12).

## Conflict of Interest

The authors declare that the research was conducted in the absence of any commercial or financial relationships that could be construed as a potential conflict of interest.

## Publisher's Note

All claims expressed in this article are solely those of the authors and do not necessarily represent those of their affiliated organizations, or those of the publisher, the editors and the reviewers. Any product that may be evaluated in this article, or claim that may be made by its manufacturer, is not guaranteed or endorsed by the publisher.
