# Supplementary figures and images for: A Chromosome Level Genome Assembly of a Winter Turnip Rape (Brassica rapa L.) to Explore the Genetic Basis of Cold Tolerance
Source: Front Plant Sci. 2022 Jul 15;13:936958. doi: 10.3389/fpls.2022.936958 (PMC9335200; doi:10.3389/fpls.2022.936958)

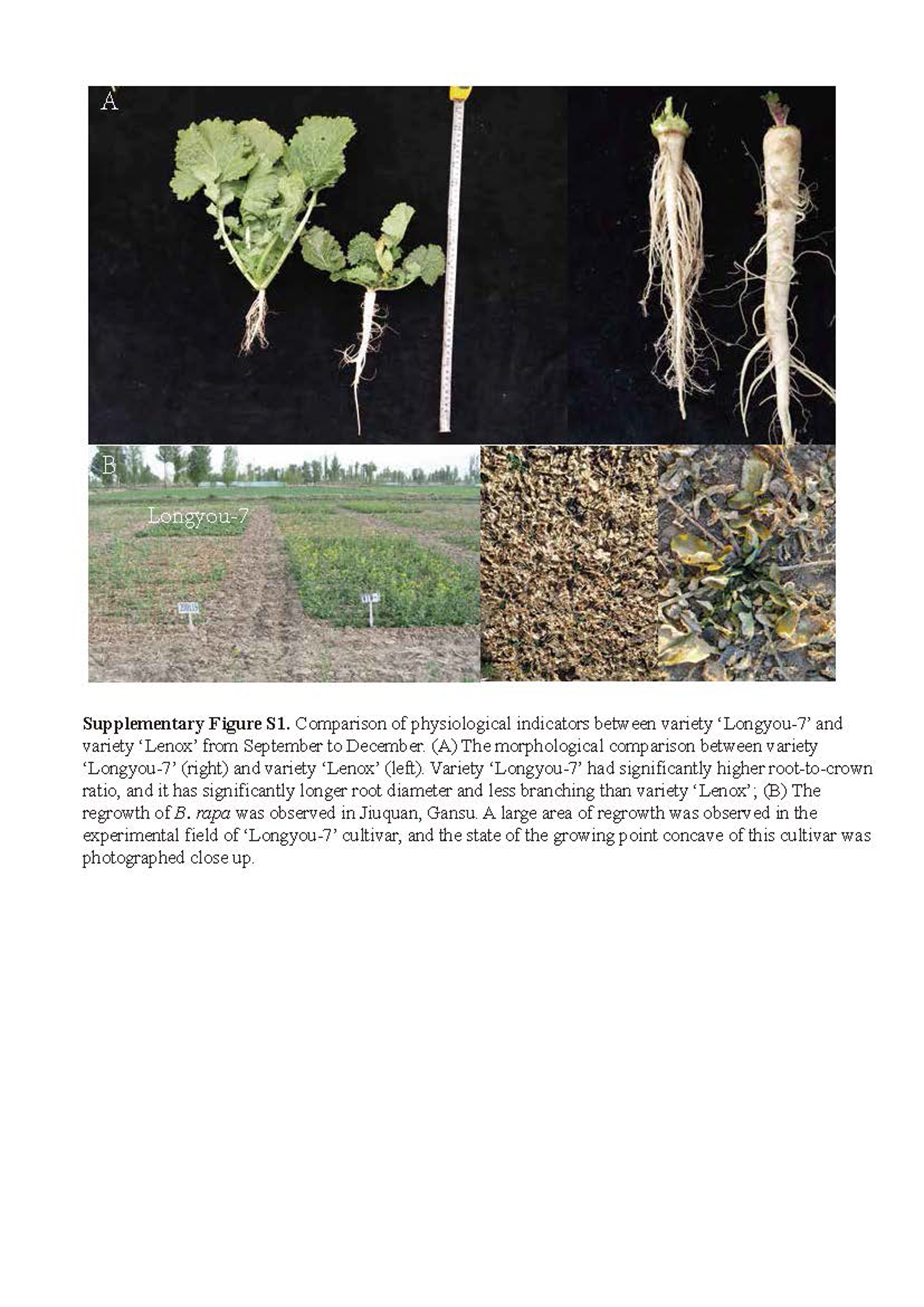

Supplement: Supplementary file 2 [file Image_1.JPEG]

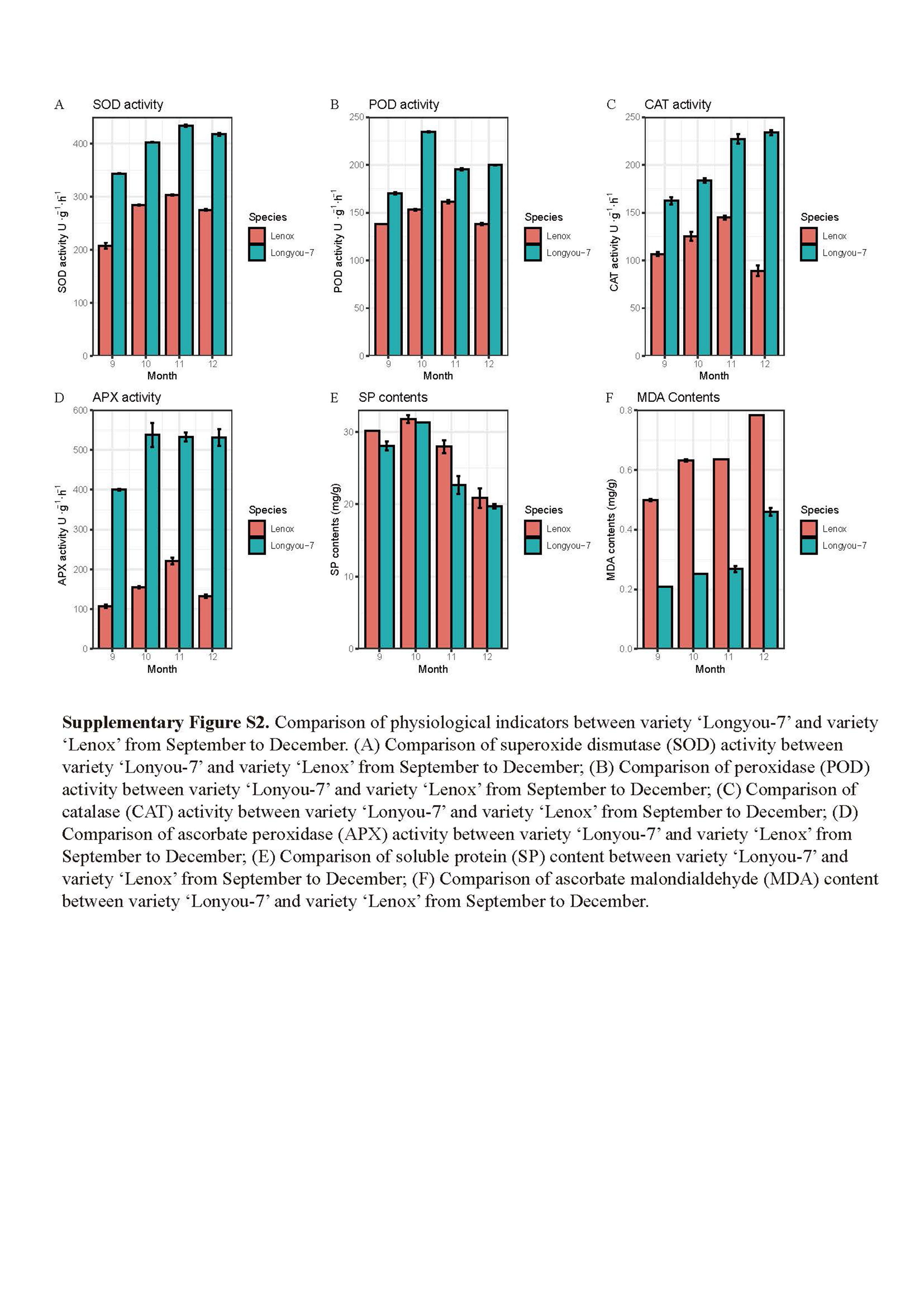

Supplement: Supplementary file 3 [file Image_2.JPEG]

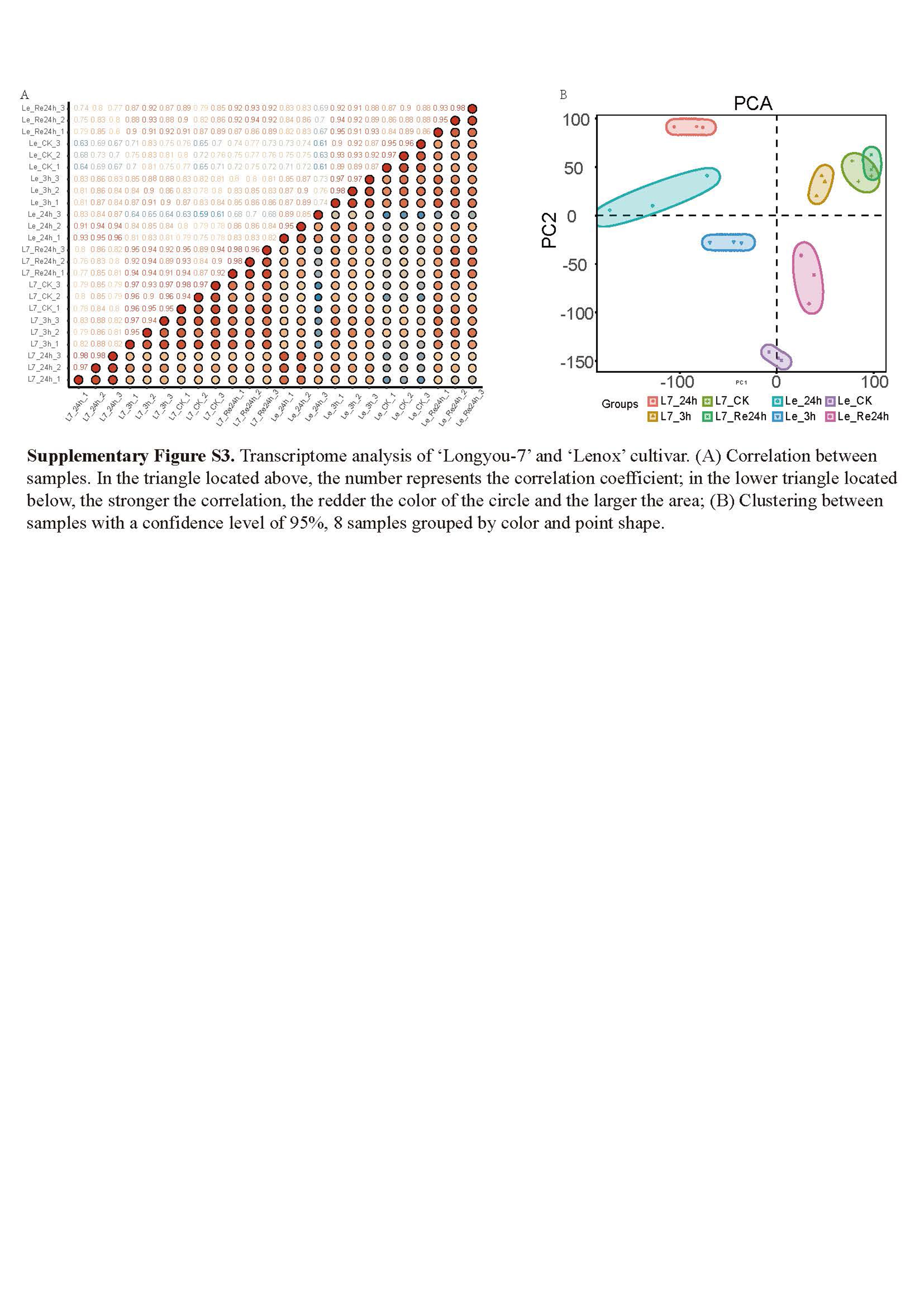

Supplement: Supplementary file 4 [file Image_3.JPEG]

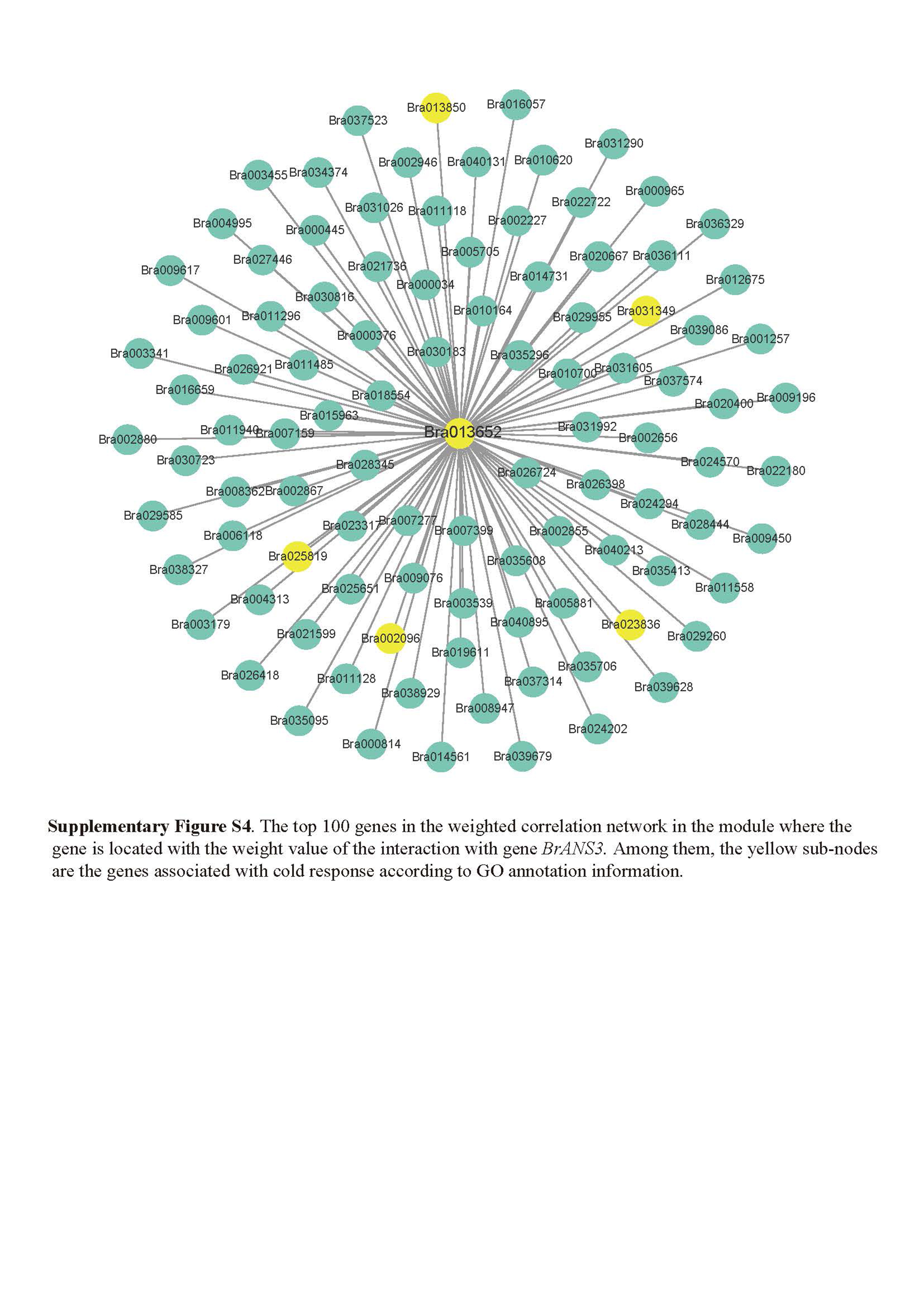

Supplement: Supplementary file 5 [file Image_4.JPEG]

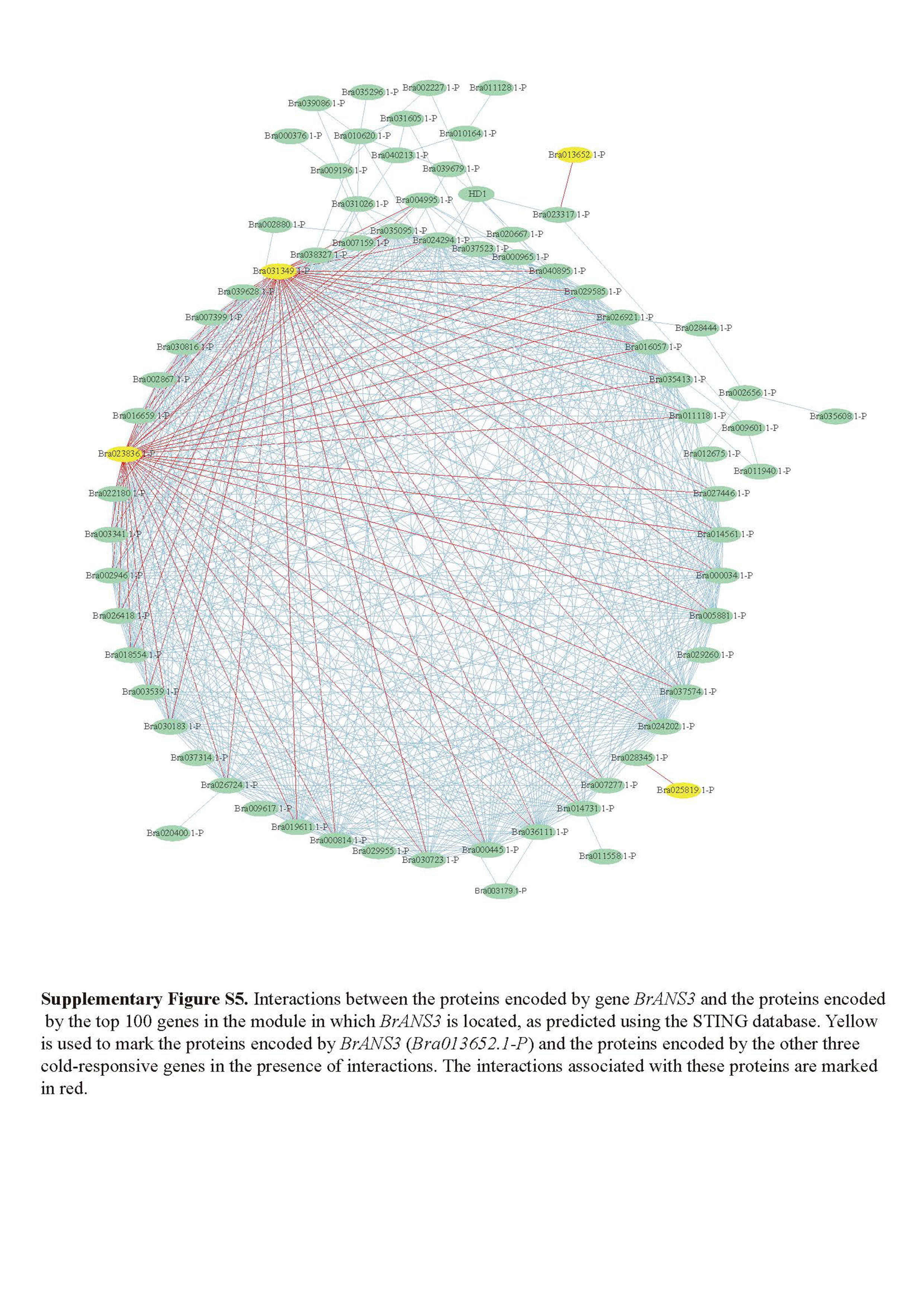

Supplement: Supplementary file 6 [file Image_5.JPEG]
